# Purcell swimmer near a wall

**DOI:** 10.1007/s11012-026-02136-0

**Published:** 2026-05-22

**Authors:** Enrico Micalizio, Marco Morandotti, Henry Shum, Marta Zoppello

**Affiliations:** 1 Department of Mathematical Sciences, Politecnico di Torino, Corso Duca degli Abruzzi, 24, Torino, 10129 Italy; 2 Department of Applied Mathematics and Waterloo Institute forNanotechnology, University of Waterloo, Waterloo, ON N2L 3G1 Canada

**Keywords:** Microswimmers, Controllability, Resistive force Theory, Wall effects

## Abstract

We study the effects of hydrodynamic interactions between a wall and a Purcell three-link swimmer in the case of motion in a plane. We extend previous theoretical studies of the three-link swimmer based on resistive-force theory by taking into account drag coefficients that are modified by the presence of a no-slip wall, considering the case where the distance to the wall is much less than the length of the swimmer and much larger than the thickness of the links. After deriving the equations of motion, we show, by means of criteria from Geometric Control Theory, that the system is locally controllable at configurations that are nearly parallel to the wall. Our result is obtained for a three-link swimmer in which the outer links have equal length and the central one differs by a factor λ>0: controllability is achieved for all values of λ. For initially horizontal swimmers, the analytical displacements obtained from Lie brackets agree with the ones computed numerically with corresponding control gaits; moreover, we find values of λ that maximize the horizontal components of displacement. Numerical calculations are used to assess the effects of an approximation to the wall-induced drag correction used in the controllability proof. Furthermore, we numerically study first- and second-order controls applied to swimmers that are slightly tilted with respect to the wall and determine which components of net displacement depend on the orientation of the swimmer relative to the wall.

## Introduction

Low-Reynolds-number locomotion has been the subject of sustained interest since Purcell’s seminal work on life at low Reynolds numbers [1]. In this regime, which is applicable to microorganisms and biomimetic microrobots, inertia is negligible and the motion of a swimmer results from non-reciprocal shape changes interacting with viscous forces. Among the paradigmatic models introduced by Purcell, the three-link swimmer now commonly referred to as the Purcell swimmer has become a key model for the theoretical study of microswimming [1], serving as a minimal yet rich system for the analysis of controllability, motion planning, and geometric aspects of locomotion.

In an unbounded planar fluid, the controllability properties of the Purcell swimmer are now well understood [2–5]. Modeling the swimmer as a kinematic control system driven by internal shape variables, several authors have shown that it is locally controllable around straight or moderately bent configurations [6, 7]. These results rely on Lie algebraic techniques and exploit the structure induced by the Stokes equations, which yield a driftless system with control vector fields associated with joint actuation. We note that these controllability results have been proved only for specific ratios λ of the length λL of the central link to the length *L* of the outer ones (λ=2 in [3], λ=1 in [4]).

By contrast, the effect of boundaries on controllability has received comparatively less attention, despite its clear physical relevance. In realistic environments, microswimmers often operate in confined or partially bounded domains, where nearby walls modify hydrodynamic interactions through no-slip boundary conditions. From a control-theoretic viewpoint, the presence of a wall could alter the resistance matrix and breaks some of the symmetries present in the unbounded case. This naturally raises the question of whether such a boundary hinders controllability by restricting the accessible motions, or whether it may instead enrich the swimmer’s dynamics and potentially enhance maneuverability.

It is commonly observed, for instance, that bacteria and spermatozoa tend to accumulate at surfaces and swim in circles near the surface [8–11]. Under steady propulsion, the symmetry-breaking of the wall induces a curved path, leading to the circular trajectory and, hence, confines the swimmer to a bounded region. Numerical and theoretical studies of swimming microorganisms have incorporated the effects of no-slip walls using image systems [12–14].

Motivated by the observation that many microorganisms use slender appendages for propulsion, the frameworks of resistive-force theory [15] and slender-body theory [16, 17] have been developed. In slender-body theory, a line distribution of Stokes flow singularities are placed along the centerline of the thin object with singularity strengths determined such that the appropriate fluid velocity boundary conditions are approximately matched on the surface of the object. By considering image singularities, the hydrodynamic effects of a no-slip wall can be incorporated [18]. In the resistive-force theory approach, a slender body is treated as a curve along which each point has drag coefficients characterizing the linear relationship between the local translational and rotational velocities (relative to the background fluid) and the forces and torques per unit length acting on the body. The coefficients are obtained from slender-body analyses and can be used to provide an approximate treatment of the wall. Expressions for the drag coefficients in the near-wall and far-field limits have been shown to agree well with numerical calculations for filaments [19].

The first objective of this paper is to address the question of controllability for a Purcell swimmer near a single planar wall and confined to motions in the plane perpendicular to the wall. Focusing on a configuration in which the swimmer is aligned and parallel to the wall, we derive the associated equation of motion using resistive-force theory with wall-induced hydrodynamic corrections [10, 18], casting it as a control system. We refer the reader to [19–23] for a list of references that take into account this wall-induced hydrodynamic correction of the drag coefficients.

Our main theoretical result (Theorem 1) shows that the presence of the wall is not an obstruction to controllability: the swimmer remains locally controllable near this aligned configuration. This demonstrates that, at least in this regime, confinement does not destroy the swimmer’s ability to generate arbitrary small motions. We stress that our controllability result is obtained for all positive values of the length ratio λ.

Beyond the analysis of controllability via Lie bracket approximations, we investigate the net displacement generated by a classical small-amplitude Purcell stroke and by a corresponding second-order stroke when the swimmer starts from a straight configuration tilted with respect to the wall. By explicitly computing the induced motion, we show that the stroke produces a systematic drift in the direction of the initial orientation, without generating any net rotation. We further establish that the presence of the wall affects the norm of the displacement which is no longer constant as in an unbounded fluid domain; this norm is maximized when the swimmer is parallel to the wall.

This behavior differs from that reported in numerical simulations and experiments on swimming spermatozoa and bacteria, where more detailed descriptions of swimming gaits and hydrodynamic interactions typically predict a reorientation and progressive alignment with the wall, often leading to surface accumulation [8, 9, 11] or, in some regimes, escape [12, 24]. Although the forward-swimming gait for the Purcell swimmer is not hydrodynamically deflected by the wall as it approaches, we show with results from a control-oriented framework based on Lie bracket computations that the swimmer can be steered both toward and away from the wall by adjusting its gait.

## Mathematical model

**Fig. 1:**
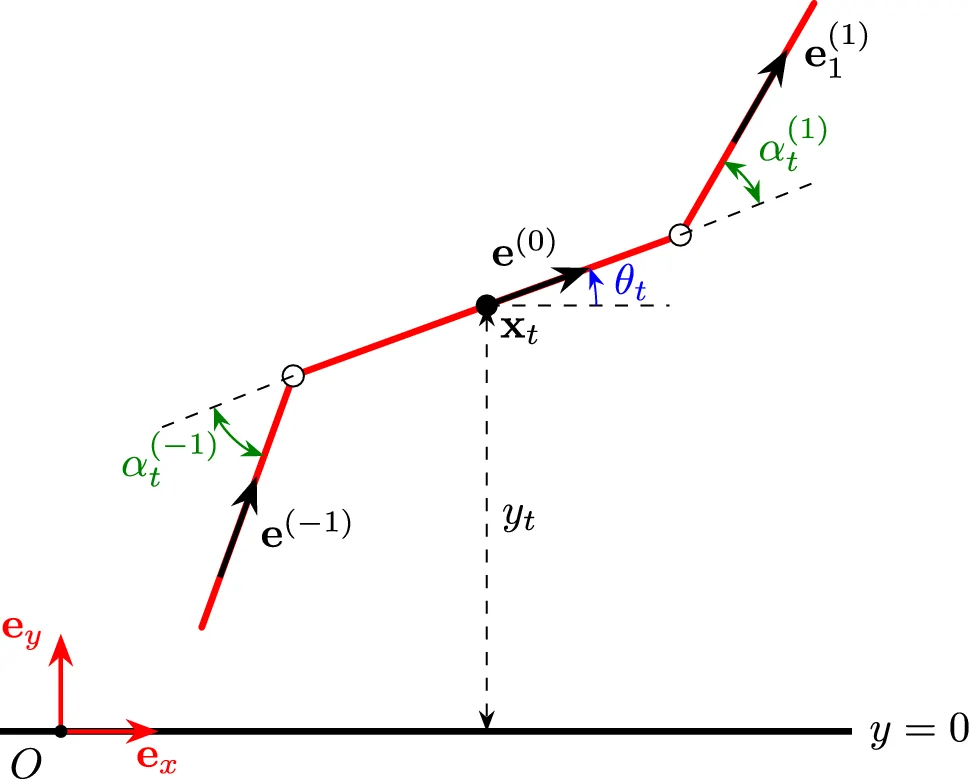
Representation of the Purcell swimmer near the wall

We consider a system composed of a three-link swimmer and a rigid wall, modeled as a straight line in our 2D model, and we adopt a reference frame in which the wall coincides with the *x*-axis. Let us denote by $y_t$ the distance, at time *t*, between the wall and the midpoint of the central link, whose position in the plane, at time *t*, is $\textbf{x}_t=(x_t,y_t)$. Moreover, the orientation of the central link with respect to the wall is described by the angle $\theta _t \in [-\pi , \pi )$, while the relative angles between the central link and the two outer links are denoted by $\alpha _t^{(\pm 1)}\in [-\pi ,\pi )$, see Fig. 1.

Thus, the triple $(x_t, y_t, \theta _t)$ describes the global position of the swimmer at time *t*, whereas the pair $\boldsymbol{\alpha }_t:=\big [\alpha _t^{(-1)}, \alpha _t^{(1)}\big ]^\top$ represents the shape. Each link has constant length $L_i$ ($i\in \{\pm 1,0\}$) and thickness *r*, and we assume that

1 $$\begin{aligned} 0 < r \ll L_i\quad \text {for every}\, i\in \{\pm 1,0\}. \end{aligned}$$

We also assume that all parts of the swimmer are far from the wall compared with *r*,

2a $$\begin{aligned} y_t\pm \frac{L_0}{2}\sin \theta _t\gg &   r,\quad \text {for every}\, t\, \text {and} \end{aligned}$$

2b $$\begin{aligned} y_t+i\biggl (\frac{L_0}{2}\sin \theta _t+ L_i\sin (\theta _t+\alpha _t^{(i)})\biggr )\gg &   r, \quad \text {for}\, i=\pm 1\, \text {and for every}\, t. \end{aligned}$$

We denote by $\textbf{e}_t^{(i)}$ the unit vector describing the direction of the *i*-th link, defined as

$$ \textbf{e}_t^{(i)} :=\begin{bmatrix} \cos \big (\theta _t + i^2\alpha _t^{(i)}\big )\\ \sin \big (\theta _t + i^2\alpha _t^{(i)}\big ) \end{bmatrix}, \qquad \text {for}\, i=-1,0,1 $$

(we can fictitiously define $\alpha _t^{(0)}=0$; its contribution is irrelevant). Finally, we can express the position of a point $\textbf{x}_t^{(i)}(s)$ on the *i*-th link

3 $$\begin{aligned} \textbf{x}_t^{(i)}(s) = \textbf{x}_t + \sigma _-^{(i)} \frac{L_0}{2}\textbf{e}_t^{(0)} + \sigma _+^{(i)} s\,\textbf{e}_t^{(i)}, \end{aligned}$$

where $s \in [0, L_i]$ denotes the distance along the *i*-th link from its hinge, and where we have defined $\sigma _\pm ^{(i)}:={{\,\textrm{sign}\,}}(i\pm \frac{1}{2})$.^1^ Taking the time derivative in (3), the velocity of the generic point in the three-link swimmer reads

4 $$\begin{aligned} \dot{\textbf{x}}_t^{(i)}(s) = \dot{\textbf{x}}_t +\sigma _-^{(i)} \frac{L_0}{2}\dot{\theta }_t\,\textbf{n}_t^{(0)} + \sigma _+^{(i)} s\,\big (\dot{\theta }_t + i^2\dot{\alpha }_t^{(i)}\big )\textbf{n}_t^{(i)}, \end{aligned}$$

where $\textbf{n}_t^{(i)} :=R_{\pi /2}\textbf{e}_t^{(i)}$ is the normal vector obtained by rotating the tangent vector $\textbf{e}_t^{(i)}$ by π/2.

Now, we aim to apply Resistive Force Theory [15] to compute the hydrodynamic force and torque densities acting on the swimmer. However, in the presence of a wall, the drag coefficients (usually denoted by $C_{\parallel }$ and $C_{\perp }$ when they are constant) depend explicitly on the distance $d_t^{(i)}(s)=y_t^{(i)}(s)$ of the point $\textbf{x}_t^{(i)}(s)$ from the wall. Of particular interest is the near-wall case in which

5 $$\begin{aligned} y_t \ll L_i\,,\quad \text {for every}\, i\in \{\pm 1,0\}\, \text {and for every}\, t. \end{aligned}$$

as the wall would have a more significant effect. By considering line integrals of Stokeslets and their image systems, Katz *et al.* [18, equation (2.7)] derived the drag coefficients for a thin cylinder parallel to a no-slip wall for motions in the directions perpendicular to the wall and parallel to the axis of the cylinder (see also [25, Section 2.7] and [19, equation (2.35)]),

$$\begin{aligned} C_{\perp } = \frac{4 \pi \mu }{\log \big (2d/r\big )-1}\qquad \text {and}\qquad C_{\parallel }= \frac{2 \pi \mu }{\log \big (2d/r\big )}, \end{aligned}$$

where μ>0 is the dynamic viscosity, under the assumption that the distance *d* to the wall is much greater than the thickness *r* of the cylinder and much smaller than the length of the cylinder. In the regime 2d/r≫1, we can assume that $C_\perp =2C_\parallel$ . We use these coefficients to define the local force and torque densities along the links of the Purcell swimmer. Taking into account the variations in distance to the wall along each link, we have

6 $$\begin{aligned} C_{\perp t}^{(i)}(s) = \frac{4 \pi \mu }{\log \big (2d_t^{(i)}(s)/r\big )} =2C_{\parallel t}^{(i)}(s), \end{aligned}$$

Consistent with the derivation of the drag coefficients [18], we impose the constraints $d_t^{(i)}(s)=y_t^{(i)}(s)\gg r$, recall (1).

In the sequel, we will omit the explicit dependence of the drag coefficients from *t*. Let $\textbf{f}^{(i)}_t(s)$ be the hydrodynamic force density acting on the point $\textbf{x}_t^{(i)}(s)$ of the *i*-th link. Resistive Force Theory yields

7 $$\begin{aligned} \begin{aligned} \!\! \textbf{f}_t^{(i)}(s)&= \big [(C_{\parallel }^{(i)}(s) - C_{\perp }^{(i)}(s))\textbf{e}_t^{(i)} \otimes \textbf{e}_t^{(i)} + C_{\perp }^{(i)}(s)\textbf{I}_2\big ]\dot{\textbf{x}}_t^{(i)} (s) \\&= C_\perp ^{(i)}(s) \bigg [\bigg (\textbf{I}_2-\frac{1}{2}\textbf{E}_t^{(i)}\bigg )\dot{\textbf{x}}_t+\sigma _+^{(i)}\,s\big (\dot{\theta }_t+i^2\dot{\alpha }_t^{(i)}\big ) \textbf{n}_t^{(i)} \\&\quad +\sigma _-^{(i)}\frac{L_0}{2}\dot{\theta }_t \bigg (\textbf{I}_2-\frac{1}{2}\textbf{E}_t^{(i)}\bigg )\textbf{n}_t^{(0)}\bigg ], \end{aligned} \end{aligned}$$

where we defined $\textbf{E}_t^{(i)} :=\textbf{e}^{(i)}_t \otimes \textbf{e}^{(i)}_t$ for notational convenience, and $\textbf{I}_2$ is the 2×2 identity matrix. Using (7) and defining $\sigma _{*}^{(i)}:={{\,\textrm{sign}\,}}(i^2-\frac{1}{2})$,^2^ the torque density about the reference point $\textbf{x}_t$ is given by

8 $$\begin{aligned} \begin{aligned} \tau _t^{(i)}(s)&= C_\perp ^{(i)}(s)\bigg [\sigma _-^{(i)}\frac{L_0}{2}\textbf{n}_t^{(0)}\cdot \bigg (\textbf{I}_2-\frac{1}{2}\textbf{E}_t^{(i)}\bigg )\dot{\textbf{x}}_t + \sigma _+^{(i)}s\, \textbf{n}_t^{(i)}\cdot \bigg (\textbf{I}_2-\frac{1}{2}\textbf{E}_t^{(i)}\bigg )\dot{\textbf{x}}_t \\&+\frac{L_0^2}{4}\dot{\theta }_t \textbf{n}_t^{(0)}\cdot \bigg (\textbf{I}_2-\frac{1}{2}\textbf{E}_t^{(i)}\bigg )\textbf{n}_t^{(0)} + \sigma _*^{(i)}\frac{L_0}{2}\dot{\theta }_t s\, \textbf{n}_t^{(i)}\cdot \bigg (\textbf{I}_2-\frac{1}{2}\textbf{E}_t^{(i)}\bigg )\textbf{n}_t^{(0)} \\&+\sigma _*^{(i)}\frac{L_0}{2} s\, \big (\dot{\theta }_t+i^2\dot{\alpha }_t^{(i)}\big ) \textbf{n}_t^{(0)}\cdot \textbf{n}_t^{(i)} +s^2\big (\dot{\theta }_t+i^2\dot{\alpha }_t^{(i)}\big ) \bigg ]. \end{aligned} \end{aligned}$$

Therefore, the total force and torque acting on each link are given by integrating (7) and (8), respectively, over $s \in [0, L_i]$, and adding up all the contributions for i=-1,0,1. Yet, since the expressions in (7) and (8) do not admit closed-form expressions when integrated with respect to *s*, we proceed by using a second-order Taylor approximation of the coefficients. This is done using the approximation $d_t^{(i)}(s) \simeq y_t$ , which is valid when the swimmer is nearly parallel to the wall so that the difference between the closest and furthest distances on any point on the swimmer to the wall is small compared with $y_t$ .

We obtain the following expression, up to second order in *s*

9a $$\begin{aligned} C^{(i)}_{\perp }(s) \simeq G^{(i)}_t + s{H^{(i)}_t} {+ s^2 K_t^{(i)}}, \end{aligned}$$

where we have defined

9b $$\begin{aligned} G^{(i)}_t:= &   \frac{2 \pi \mu }{\ell _t}\bigg [2 - \frac{\sigma _-^{(i)} L_0 \sin (\theta _t)}{y_t\ell _t} {+\frac{L_0^2}{4} \frac{\sin ^2(\theta _t)}{y_t\ell _t^2}\bigg (1+\frac{2}{\ell _t}\bigg )}\bigg ], \end{aligned}$$

9c $$\begin{aligned} {H^{(i)}_t}:= &   {- \frac{2 \pi \mu \sin \big (\theta _t + i^2 \alpha ^{(i)}_t\big )}{y_t\ell _t^{2}}\, \bigg [2\sigma _+^{(i)} - \frac{\sigma _*^{(i)}\,L_0\sin (\theta _t)}{y_t}\bigg (1+\frac{2}{\ell _t}\bigg )\bigg ]}, \end{aligned}$$

9d $$\begin{aligned} {K^{(i)}_t}&{:=}&\frac{2 \pi \mu \sigma _+^{(i)} \sin ^2 \big (\theta _t + i^2 \alpha ^{(i)}_t\big )}{y_t^{2}\ell _t^{2}} \bigg (1 + \frac{2}{\ell _t}\bigg ), \end{aligned}$$

with $\ell _t:=\log (2 y_t/r)$. Thanks to (9), we can integrate (7) and (8) with respect to $s\in [0, L_i]$ (for i=±1,0) and obtain analytic expressions. The resulting total force and torque exerted by the fluid on the swimmer are then given by

10a $$\begin{aligned} \textbf{F}_t= &   \sum _{i = -1}^1 \int _0^{L_i} \textbf{f}_t^{(i)}(s) \,\textrm{d} s = \textbf{A}_t \dot{\textbf{x}}_t + \textbf{B}_t \dot{\boldsymbol{\alpha }}_t + \boldsymbol{\gamma }_t \dot{\theta }_t, \end{aligned}$$

10b $$\begin{aligned} T_t= &   \sum _{i = -1}^1 \int _0^{L_i} \tau _t^{(i)}(s) \,\textrm{d}s = \boldsymbol{\gamma }_t \cdot \dot{\textbf{x}}_t + \textbf{b}_t \cdot \dot{\boldsymbol{\alpha }}_t + \delta _t\dot{\theta }_t. \end{aligned}$$

In (10), we have defined the 2×2 matrices $\textbf{A}_t$ and $\textbf{B}_t$ by

$$\begin{aligned} \textbf{A}_t:= &   \sum _{i=-1}^1 \bigg (G_t^{(i)}L_i+{H_t}^{(i)}\frac{L_i^2}{2}+{K_t^{(i)}\frac{L_i^3}{3}}\bigg )\bigg (\textbf{I}_2-\frac{1}{2}\textbf{E}_t^{(i)}\bigg ), \\ \textbf{B}_t:= &   \bigg [-\bigg (G_t^{(-1)}\frac{L_{-1}^2}{2}+{H_t}^{(-1)}\frac{L_{-1}^3}{3}{+K_t^{(-1)}\frac{L_{-1}^4}{4}}\bigg )\textbf{n}_t^{(-1)} \bigg | \\  &   \bigg (G_t^{(1)}\frac{L_{1}^2}{2}+{H_t}^{(1)}\frac{L_{1}^3}{3}{+K_t^{(1)}\frac{L_1^4}{4}}\bigg )\textbf{n}_t^{(1)}\bigg ] \end{aligned}$$

(notice that $\textbf{A}_t$ is symmetric), the vectors $\boldsymbol{\gamma }_t$ and $\textbf{b}_t$ by

$$\begin{aligned} \boldsymbol{\gamma }_t:= &   \sum _{i=-1}^1 \bigg [\bigg (G_t^{(i)}\frac{L_i^2}{2}+{H_t}^{(i)}\frac{L_{i}^3}{3}{+K_t^{(i)}\frac{L_{i}^4}{4}}\bigg )\sigma _+^{(i)}\textbf{n}_t^{(i)} \\  &   +\frac{L_0}{2}\sigma _-^{(i)}\bigg (G_t^{(i)}L_i+{H_t}^{(i)}\frac{L_{i}^3}{3}{+K_t^{(i)}\frac{L_{i}^4}{4}}\bigg ) \bigg (\textbf{I}_2-\frac{1}{2}\textbf{E}_t^{(i)}\bigg ) \textbf{n}_t^{(0)}\bigg ],\quad \\ \textbf{b}_t:= &   \Bigg [ \frac{L_0}{2}\bigg (G_t^{(-1)}\frac{L_{-1}^2}{2} + {H_t}^{(-1)}\frac{L_{-1}^3}{3}{+K_t^{(-1)}\frac{L_{-1}^4}{4}} \bigg )\textbf{n}_t^{(0)} \cdot \textbf{n}_t^{(-1)} \\  &   + \bigg (G_t^{(-1)}\frac{L_{-1}^3}{3} + {H_t}^{(-1)} \frac{L_{-1}^4}{4}{+K_t^{(-1)}\frac{L_{-1}^5}{5}} \bigg )\bigg | \\  &   \frac{L_0}{2}\bigg (G_t^{(1)}\frac{L_{1}^2}{2} + {H_t}^{(1)}\frac{L_{1}^3}{3}{+K_t^{(1)}\frac{L_{1}^4}{4}} \bigg )\textbf{n}_t^{(0)} \cdot \textbf{n}_t^{(1)} + \bigg (G_t^{(1)}\frac{L_{1}^3}{3} + {H_t}^{(1)} \frac{L_{1}^4}{4} {+K_t^{(1)}\frac{L_{1}^5}{5}}\bigg )\bigg ], \end{aligned}$$

and the scalar $\delta _t$ by

$$\begin{aligned} \begin{aligned} \delta _t :=&\sum _{i = -1}^1 \Bigg [ \frac{L_0^2}{4}\bigg (G_t^{(i)}L_i + {H_t}^{(i)} \frac{L_i^2}{2}{+K_t^{(i)}\frac{L_i^3}{3}}\bigg ) \textbf{n}_t^{(0)}\cdot \bigg (\textbf{I}_2-\frac{1}{2}\textbf{E}_t^{(i)}\bigg )\textbf{n}_t^{(0)} \\&\phantom {\sum _{i = -1}^1 \Bigg [} + \frac{L_0}{2}\sigma _*^{(i)} \bigg (G_t^{(i)}\frac{L_i^2}{2} + {H_t}^{(i)} \frac{L_i^3}{3}{+K_t^{(i)}\frac{L_i^4}{4}} \bigg ) \textbf{n}_t^{(0)}\cdot \bigg (2\textbf{I}_2-\frac{1}{2}\textbf{E}_t^{(i)}\bigg )\textbf{n}_t^{(0)} \\&\phantom {\sum _{i = -1}^1 \Bigg [} + G_t^{(i)}\frac{L_i^3}{3} + {H_t}^{(i)} \frac{L_i^4}{4}{+K_t^{(i)}\frac{L_i^5}{5}} \bigg ]. \end{aligned} \end{aligned}$$

Finally, from (10), the equations of motion can be written compactly as

11 $$\begin{aligned} \!\!\!\!\! \textbf{0}=\begin{bmatrix} \textbf{F}_t \\ T_t \end{bmatrix} = \begin{bmatrix} \textbf{A}_t &  \boldsymbol{\gamma }_t &  \textbf{B}_t \\ \boldsymbol{\gamma }_t^\top &  \delta _t &  \textbf{b}_t^\top \end{bmatrix}\!\! \begin{bmatrix} \dot{\textbf{x}}_t \\ \dot{\theta }_t \\ \dot{\boldsymbol{\alpha }}_t \end{bmatrix} = \begin{bmatrix} (\textbf{A}_t)_{11} &  (\textbf{A}_t)_{12} &  (\boldsymbol{\gamma }_t)_1 &  (\textbf{B}_t)_{11} &  (\textbf{B}_t)_{12} \\ (\textbf{A}_t)_{21} &  (\textbf{A}_t)_{22} &  (\boldsymbol{\gamma }_t)_2 &  (\textbf{B}_t)_{21} &  (\textbf{B}_t)_{22} \\ (\boldsymbol{\gamma }_t)_1 &  (\boldsymbol{\gamma }_t)_2 &  \delta _t &  (\textbf{b}_t)_1 &  (\textbf{b}_t)_2 \end{bmatrix}\!\! \begin{bmatrix} \dot{x}_t \\ \dot{y}_t \\ \dot{\theta }_t \\ \dot{\alpha }_t^{(-1)} \\ \dot{\alpha }_t^{(1)} \end{bmatrix}. \end{aligned}$$

## Controllability

To study the controllability of the system, we employ tools from Geometric Control Theory. Starting from equation (11), we introduce two control functions $t\mapsto u_t^{(\pm 1)}$ corresponding to the shape changes governed by $\dot{\alpha }_t^{(\pm 1)}$, respectively. Eventually, we will define the associated control fields $\textbf{h}_t^{(\pm 1)}$ which will be used to compute Lie brackets and analyze the controllability of the system.

By introducing

$$ \textbf{M}_t :=\begin{bmatrix} \textbf{A}_t &  \boldsymbol{\gamma }_t \\ \boldsymbol{\gamma }_t^\top &  \delta _t \end{bmatrix},\quad \textbf{g}^{(-1)}_t :=\begin{bmatrix} (\textbf{B}_t)_{11} \\ (\textbf{B}_t)_{21} \\ (\textbf{b}_t)_1 \end{bmatrix}, \quad \text {and}\quad \textbf{g}^{(1)}_t :=\begin{bmatrix} (\textbf{B}_t)_{12} \\ (\textbf{B}_t)_{22} \\ (\textbf{b}_t)_2 \end{bmatrix}, $$

and the control inputs $[u_t^{(-1)}, u_t^{(1)}]^\top :=[\dot{\alpha }_t^{(-1)}, \dot{\alpha }_t^{(1)}]^\top$, the equations of motion (11) can be rewritten as

12 $$\begin{aligned} \begin{bmatrix} \textbf{M}_t&  \textbf{0}_{3 \times 2} \\ \textbf{0}_{2 \times 3} &  \textbf{I}_{2} \end{bmatrix} \begin{bmatrix} \dot{\textbf{x}}_t \\ \dot{\theta }_t \\ \dot{\boldsymbol{\alpha }}_t \end{bmatrix} = - \begin{bmatrix} \textbf{g}_t^{(-1)} &  \textbf{g}_t^{(1)} \\ 1 &  0 \\ 0 &  1 \end{bmatrix} \begin{bmatrix} u_t^{(-1)} \\ u_t^{(1)} \end{bmatrix}. \end{aligned}$$

The matrix $\textbf{M}_t$ is usually referred to as the *grand resistance matrix*; it is symmetric and invertible around the “flat” ($\alpha _t^{(\pm 1)}=0$) horizontal ($\theta _t=0$) configuration, at which we have $\det \textbf{M}_t=8\pi ^3\mu ^3(L_{-1}+L_0+L_1)^5/3\ell _t^3\ne 0$. By continuity of the determinant with respect to the matrix entries, $\det \textbf{M}_t$ remains different from zero also at configurations $\theta _t,\alpha _t^{(\pm 1)}\approx 0$. This allows us to solve (12) for the position and shape variables and write it as a nonlinear driftless affine control system

13 $$\begin{aligned} \begin{bmatrix} \dot{\textbf{x}}_t \\ \dot{\theta }_t\\ \dot{\boldsymbol{\alpha }}_t \end{bmatrix} \;=\; \textbf{h}_t^{(-1)}u_t^{(-1)} + \textbf{h}_t^{(1)}u_t^{(1)} \end{aligned}$$

where the control vector fields $\textbf{h}_t^{(\pm 1)}$ are defined by

$$ \textbf{h}_t^{(-1)} :=\begin{bmatrix} -\textbf{M}_t^{-1}\textbf{g}^{(-1)}_t \\ 1\\ 0 \end{bmatrix}, \qquad \textbf{h}_t^{(1)} :=\begin{bmatrix} -\textbf{M}_t^{-1}\textbf{g}^{(1)}_t \\ 0\\ 1 \end{bmatrix}. $$

We are now ready to investigate the local controllability of (13). To simplify computations, we suppose that the outer links have length $L_{-1}=L_1=L$ and that the central one has length $L_0=\lambda L$, for some λ>0, provided that (1) and () are respected.

### Theorem 1

Let L,r,λ>0 and let $L_{\pm 1}=L$, $L_0=\lambda L$; let us assume that (1) and () hold true. Then control system (13) is locally controllable around an aligned configuration parallel to the wall, *i.e.*, for $\theta _t,\alpha _t^{(\pm 1)}\approx 0$.

### Proof

In order to prove the local controllability of the system (13) we resort to the Chow–Rashewskii theorem [26, Theorem 5.9], which states that a sufficient condition for local controllability at a certain configuration is that the Lie algebra generated by the control vector fields at that configuration has dimension equal to the dimension of the tangent space of the overall system. In our specific case, we have to show that the control vector fields $\textbf{h}_t^{(\pm 1)}$ and their iterated Lie brackets at $(x_t,y_t,\theta _t, \alpha _t^{(-1)}, \alpha _t^{(1)} )=(x_t,y_t,0,0,0)=:\textbf{s}_t^0$ generate a space of dimension five. Through explicit computations, we obtain

$$\begin{aligned} \textbf{h}_t^1(\lambda ):=\textbf{h}_t^{(-1)} \big |_{\textbf{s}_t^0} = \begin{bmatrix} 0 \\ \displaystyle \frac{L}{ 2 \lambda + 4}\\ \displaystyle \frac{ - 3 \lambda -4}{(\lambda + 2)^3}\\ 1 \\ 0 \end{bmatrix},\qquad \textbf{h}_t^2(\lambda ):=\textbf{h}_t^{(1)} \big |_{\textbf{s}_t^0} = \begin{bmatrix} 0 \\ \displaystyle -\frac{L}{2 \lambda + 4}\\ \displaystyle \frac{- 3 \lambda -4}{(\lambda + 2)^3}\\ 0 \\ 1 \end{bmatrix}, \end{aligned}$$

14 $$\begin{aligned} \textbf{h}_t^3(\lambda ):=[\textbf{h}_t^{(-1)}, \textbf{h}_t^{(1)}] \big |_{\textbf{s}_t^0} = \begin{bmatrix} \displaystyle \frac{L \lambda (2 \lambda +3)}{(\lambda + 2)^4}\\ 0 \\ 0 \\ 0 \\ 0 \end{bmatrix}, \end{aligned}$$

15 $$\begin{aligned} \begin{aligned}&{\textbf{h}_t^{4}(\lambda ):=[\textbf{h}_t^{(1)},[\textbf{h}_t^{(-1)}, \textbf{h}_t^{(1)}]] \big |_{\textbf{s}_t^0} = \begin{bmatrix} \displaystyle -\frac{L^2 \lambda (4 \lambda ^4 +7\lambda ^3-18\lambda ^2-52\lambda -34 )}{6 (\lambda + 2)^7 \ell _t y_t} \\ \displaystyle \frac{L\lambda ^2(3\lambda ^3+20\lambda ^2 + 38\lambda + 22)}{2(\lambda + 2)^7 }\\ \displaystyle \frac{3\lambda }{(\lambda + 2)^{4}}\\ 0\\ 0 \end{bmatrix}}, \end{aligned} \end{aligned}$$

$$\begin{aligned} \begin{aligned}&{\textbf{h}_t^{5}(\lambda ):=[\textbf{h}_t^{(-1)},[\textbf{h}_t^{(-1)}, \textbf{h}_t^{(1)}]] \big |_{\textbf{s}_t^0} = \begin{bmatrix} \displaystyle \frac{L^2 \lambda (4 \lambda ^4 +7\lambda ^3-18\lambda ^2-52\lambda -34 )}{6 (\lambda + 2)^7 \ell _t y_t} \\ \displaystyle \frac{L\lambda ^2(3\lambda ^3+20\lambda ^2 + 38\lambda + 22)}{2(\lambda + 2)^7 }\\ \displaystyle -\frac{3\lambda }{(\lambda + 2)^{4}}\\ 0\\ 0 \end{bmatrix}}. \end{aligned} \end{aligned}$$

Finally, we can compute the determinant of the resulting 5×5 matrix, which reads

$$ D(\lambda ) :=\textrm{det}\big (\textbf{h}_t^{1}(\lambda )\,|\,\textbf{h}_t^{2}(\lambda ) \,|\, \textbf{h}_t^{3}(\lambda ) \,|\, \textbf{h}_t^{4}(\lambda ) \,|\, \textbf{h}_t^{5}(\lambda )\big ) = {-\frac{3L^2 \lambda ^4 (2\lambda +3)(3\lambda ^3 + 20\lambda ^2 + 38\lambda + 22)}{(\lambda + 2)^{15}}}, $$

which is negative for every λ>0.

This proves the local controllability of the system at the configuration $\textbf{s}_t^0$ for every λ>0.

For tilted configurations at which $\theta _0\ne 0$ is sufficiently small in magnitude, the system remains locally controllable. Indeed, since the vector fields and their Lie brackets are real analytic, their determinant is a continuous function. Therefore, the determinant in the configuration $(x_0,y_0,\theta _0,0,0)$ will remain different from zero, proving local controllability. □

### Remark 1

It is well known (see, e.g., [27]) that the displacement of the system which starts in the aligned configuration parallel to the wall after a periodic control stroke with piecewise constant controls is given by a multiple of the first order bracket $\textbf{h}_{t}^3(\lambda )$. From (14), it emerges that this results in a displacement parallel to the wall, independently from the distance of the swimmer from the wall, for all values of λ. Notice that this independence is not linked to the symmetric design of the three-link swimmer: indeed, if the three links all have different lengths, the Lie bracket at the aligned configuration parallel to the wall is given by

$$ \textbf{h}_t^{3}(L_{-1},L_0,L_1)= \begin{bmatrix} \displaystyle \frac{L_0 L_1 L_{-1} \bigl (L_1 (L_0 + L_1) + (L_0 + L_1) L_{-1} + L_{-1}^2\bigr )}{(L_0 + L_1 + L_{-1})^4}\\ 0 \\ 0 \\ 0 \\ 0 \end{bmatrix}, $$

and notice that $\textbf{h}_t^3(L,\lambda L,L)=\textbf{h}_t^3(\lambda )$. Therefore, we can conclude that if the swimmer is parallel to the wall, after making a small periodic control stroke with piecewise controls, it will move parallel to the wall, regardless of the length of the links.

## Numerical simulations

In this section, we take advantage of numerical tools to solve the control system (13). The approximation (9a) of the drag coefficients, which was a simplification that allowed analytical controllability results to be obtained, is not necessary for the numerical solutions. We begin with a sensitivity analysis to determine whether this approximation has a significant effect on the dynamics and then present additional characterizations of the system using the nonlinear coefficients (6), including the dependence of the spatial and angular displacements on the link length ratio λ introduced in Theorem 1.

We solve the control system (13) in MATLAB using the ode45 function, which is an implementation of the Runge–Kutta–Fehlberg 4(5) numerical ODE solver. The controls $u_t^{(-1)}$ and $u_t^{(1)}$ are defined as piecewise constant functions with an amplitude parameter ξ>0 such that the net displacement after one period of the control corresponds to the defined vector fields $\textbf{h}^1_t, \ldots , \textbf{h}^5_t$, respectively, in the limit of $\xi \rightarrow 0$.

For numerical simulations corresponding to the vector field $\textbf{h}^3_t=[\textbf{h}^1_t,\textbf{h}^2_t]$, which results in swimming along the direction of the links, we start from the straight configuration $\alpha ^{(-1)}_0=\alpha ^{(1)}_0=0$ and apply the 4-periodic controls

16 $$\begin{aligned} u_t^{(-1)} = {\left\{ \begin{array}{ll} \xi , &  t \;\textrm{mod}\; 4 \in [0, 1),\\ 0, &  t \;\textrm{mod}\; 4 \in [1 , 2),\\ -\xi , &  t \;\textrm{mod}\; 4 \in [2 , 3),\\ 0, &  t \;\textrm{mod}\; 4 \in [3, 4), \end{array}\right. }\hspace{1cm} u_t^{(1)} = {\left\{ \begin{array}{ll} 0, &  t \;\textrm{mod}\; 4 \in [0 , 1),\\ \xi , &  t \;\textrm{mod}\; 4 \in [1 , 2),\\ 0, &  t \;\textrm{mod}\; 4 \in [2 , 3),\\ -\xi , &  t \;\textrm{mod}\; 4 \in [3 , 4). \end{array}\right. } \end{aligned}$$

**Fig. 2:**
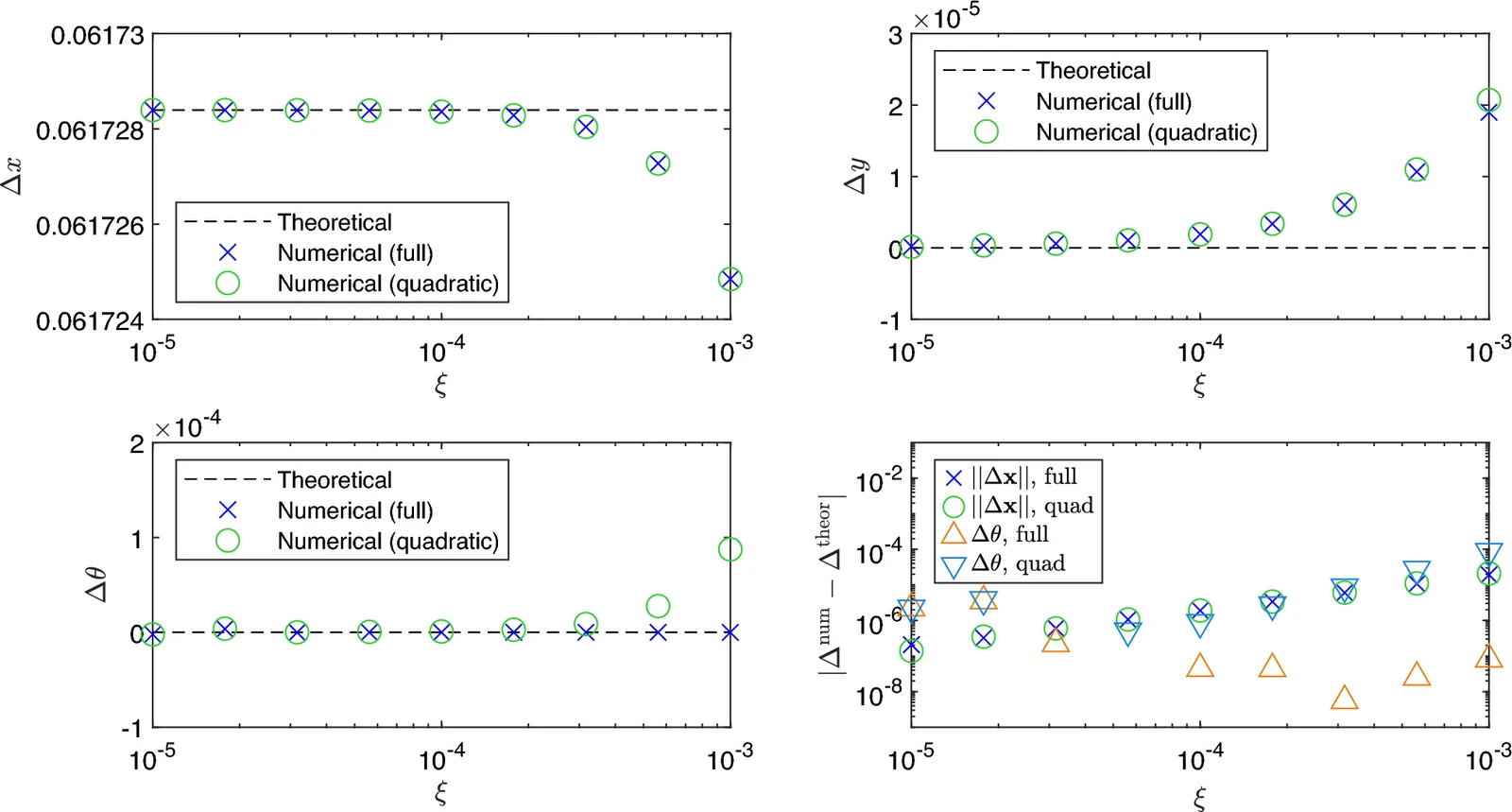
Normalized displacements Δx, Δy, and $\Delta \theta$ for a swimming stroke corresponding to the vector field $\textbf{h}^3_t$ . The values computed numerically using the full drag coefficients (6) and the quadratic approximation (9) are compared as functions of stroke amplitude ξ, and the corresponding theoretical values for the limit $\xi \rightarrow 0$ from the vector field $\textbf{h}^3_t$  are indicated. The initial state is $(x_0,y_0,\theta _0,\alpha ^{(-1)}_0,\alpha ^{(1)}_0) = (0,10^{-2},0,0,0)$. The geometrical parameters used are L=1, λ=1, $r=10^{-4}$. The theoretical values from $\textbf{h}^3_t$ are $\Delta x\approx 0.0617, \Delta y = \Delta \theta = 0$. At $\xi =10^{-4.25}$, we found $|\Delta \theta ^\textrm{num}-\Delta \theta ^\textrm{theor}|=0$ for the full coefficients so this data point is not shown on the logarithmic scale

The normalized displacements per cycle are given by (see, *e.g.*, [26, Section 1.4] or [27, Section 3.2])

17 $$\begin{aligned} \boldsymbol{\Delta }= \begin{bmatrix} \Delta x \\ \Delta y \\ \Delta \theta \\ \Delta \alpha ^{(-1)}\\ \Delta \alpha ^{(1)} \end{bmatrix}:= &   \frac{1}{\xi ^2} \begin{bmatrix} x_4 - x_0\\ y_4 - y_0\\ \theta _4 - \theta _0\\ \alpha ^{(-1)}_4 - \alpha ^{(-1)}_0\\ \alpha ^{(1)}_4 - \alpha ^{(1)}_0 \end{bmatrix} = \textbf{h}^3_0 + \begin{bmatrix} O(\xi )\\ O(\xi ) \\ O(\xi ) \\ 0 \\ 0\end{bmatrix} \end{aligned}$$

as $\xi \rightarrow 0$. In Fig. 2, the *x*, *y*, and θ components of $\boldsymbol{\Delta }$ are plotted along with their corresponding components of $\textbf{h}^3_t$ starting from a straight configuration of the links parallel to the wall. The numerical results converge to the asymptotic result as $\xi \rightarrow 0$. We note that for finite ξ there is a slight drift away from the wall (Δy>0) when the swimmer is initially parallel to the wall. The magnitude of $\Delta \theta$ computed using the full drag coefficients is insignificant for all tested ξ. We note, however, that the error (compared with the asymtotic result) in $\Delta \theta$ computed with the full coefficient formula increases when ξ decreases below about $10^{-3.5}$. We attribute this to numerical errors resulting from imperfect cancellation of small displacements over the periodic cycle of motion.

To support our theoretical controllability result, we also numerically simulate controls corresponding to the second-order vector field $\textbf{h}^4_t=[\textbf{h}^2_t,[\textbf{h}^1_t,\textbf{h}^2_t]]$, which results in swimming along the direction perpendicular to the links and a rotation of the swimmer. Starting from the straight configuration $\alpha ^{(-1)}_0=\alpha ^{(1)}_0=0$, we apply the 10-periodic controls

18 $$\begin{aligned} u_t^{(-1)} = {\left\{ \begin{array}{ll} 0, &  t \;\textrm{mod}\; 10 \in [0,1),\\ \xi , &  t \;\textrm{mod}\; 10 \in [1,2),\\ 0, &  t \;\textrm{mod}\; 10 \in [2,3),\\ -\xi , &  t \;\textrm{mod}\; 10 \in [3,4),\\ 0, &  t \;\textrm{mod}\; 10 \in [4,5),\\ 0, &  t \;\textrm{mod}\; 10 \in [5,6),\\ 0, &  t \;\textrm{mod}\; 10 \in [6,7),\\ \xi , &  t \;\textrm{mod}\; 10 \in [7,8),\\ 0, &  t \;\textrm{mod}\; 10 \in [8,9),\\ -\xi , &  t \;\textrm{mod}\; 10 \in [9,10), \end{array}\right. }\hspace{1cm} u_t^{(1)} = {\left\{ \begin{array}{ll} \xi , &  t \;\textrm{mod}\; 10 \in [0,1),\\ 0, &  t \;\textrm{mod}\; 10 \in [1,2),\\ \xi , &  t \;\textrm{mod}\; 10 \in [2,3),\\ 0, &  t \;\textrm{mod}\; 10 \in [3,4),\\ -\xi , &  t \;\textrm{mod}\; 10 \in [4,5),\\ -\xi , &  t \;\textrm{mod}\; 10 \in [5,6),\\ \xi , &  t \;\textrm{mod}\; 10 \in [6,7),\\ 0, &  t \;\textrm{mod}\; 10 \in [7,8),\\ -\xi , &  t \;\textrm{mod}\; 10 \in [8,9),\\ 0, &  t \;\textrm{mod}\; 10 \in [9,10). \end{array}\right. } \end{aligned}$$

**Fig. 3:**
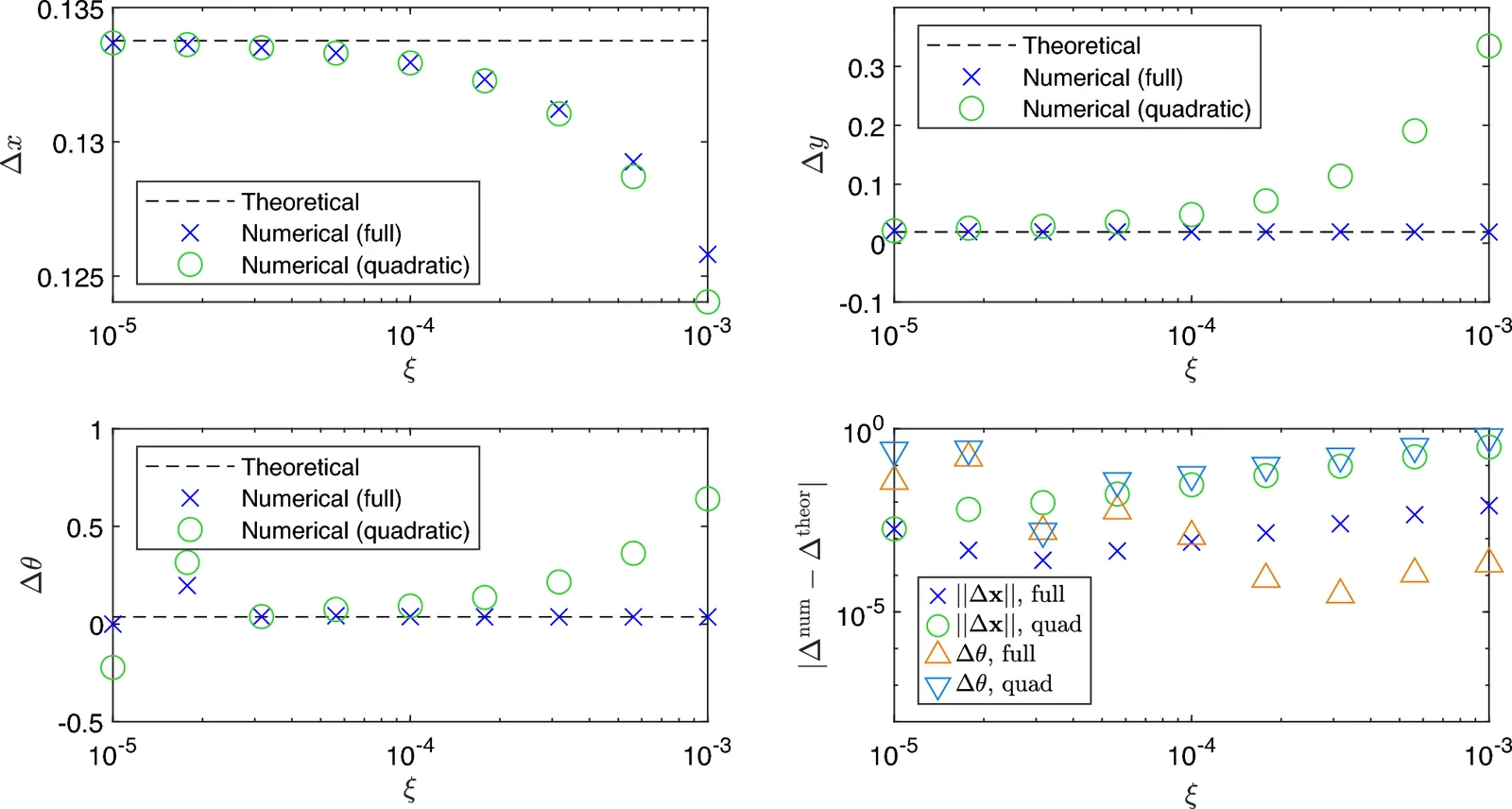
Normalized displacements Δx, Δy, and $\Delta \theta$ for a swimming stroke corresponding to the vector field $\textbf{h}^4_t$ . The values computed numerically using the full drag coefficients (6) and the quadratic approximation (9) are compared as functions of stroke amplitude ξ, and the corresponding theoretical values for the limit $\xi \rightarrow 0$ from the vector field $\textbf{h}^4_t$  are indicated. The initial state is $(x_0,y_0,\theta _0,\alpha ^{(-1)}_0,\alpha ^{(1)}_0) = (0,10^{-2},0,0,0)$. The geometrical parameters used are L=1, λ=1, $r=10^{-4}$. The theoretical values from $\textbf{h}^4_t$ are $(\Delta x, \Delta y, \Delta \theta ) \approx (0.1338,0.0190,0.0370)$

We define the normalized displacements per cycle for this family of controls by

19 $$\begin{aligned} \boldsymbol{\Delta }= \begin{bmatrix} \Delta x \\ \Delta y \\ \Delta \theta \\ \Delta \alpha ^{(-1)}\\ \Delta \alpha ^{(1)} \end{bmatrix}:= &   \frac{1}{\xi ^3} \begin{bmatrix} x_{10} - x_0\\ y_{10} - y_0\\ \theta _{10} - \theta _0\\ \alpha ^{(-1)}_{10} - \alpha ^{(-1)}_0\\ \alpha ^{(1)}_{10} - \alpha ^{(1)}_0 \end{bmatrix} = \textbf{h}^4_0 + \begin{bmatrix} O(\xi )\\ O(\xi ) \\ O(\xi ) \\ 0 \\ 0\end{bmatrix} \end{aligned}$$

as $\xi \rightarrow 0$. Numerically evaluated normalized displacements are shown in Fig. 3. Discrepancies between the finite-ξ computations and the theoretical asymptotic limit are generally larger than those found for the $\textbf{h}^3_t$ vector field. Since the second-order control yields a much smaller $O(\xi ^3)$ net displacement before normalization, computations are more susceptible to round-off and other numerical errors.

Note that $\textbf{h}_t^5$ has the same components, possibly up to a sign, of $\textbf{h}_t^4$ , so we do not present numerical results for it. The comparisons done so far and presented in Figs. 2 and 3 show a good agreement between numerical solutions using the full resistive coefficients and Lie bracket calculation which were based on the quadratic approximation (9), hence we expect the local controllability result to hold for the full coefficients (6).

**Fig. 4:**
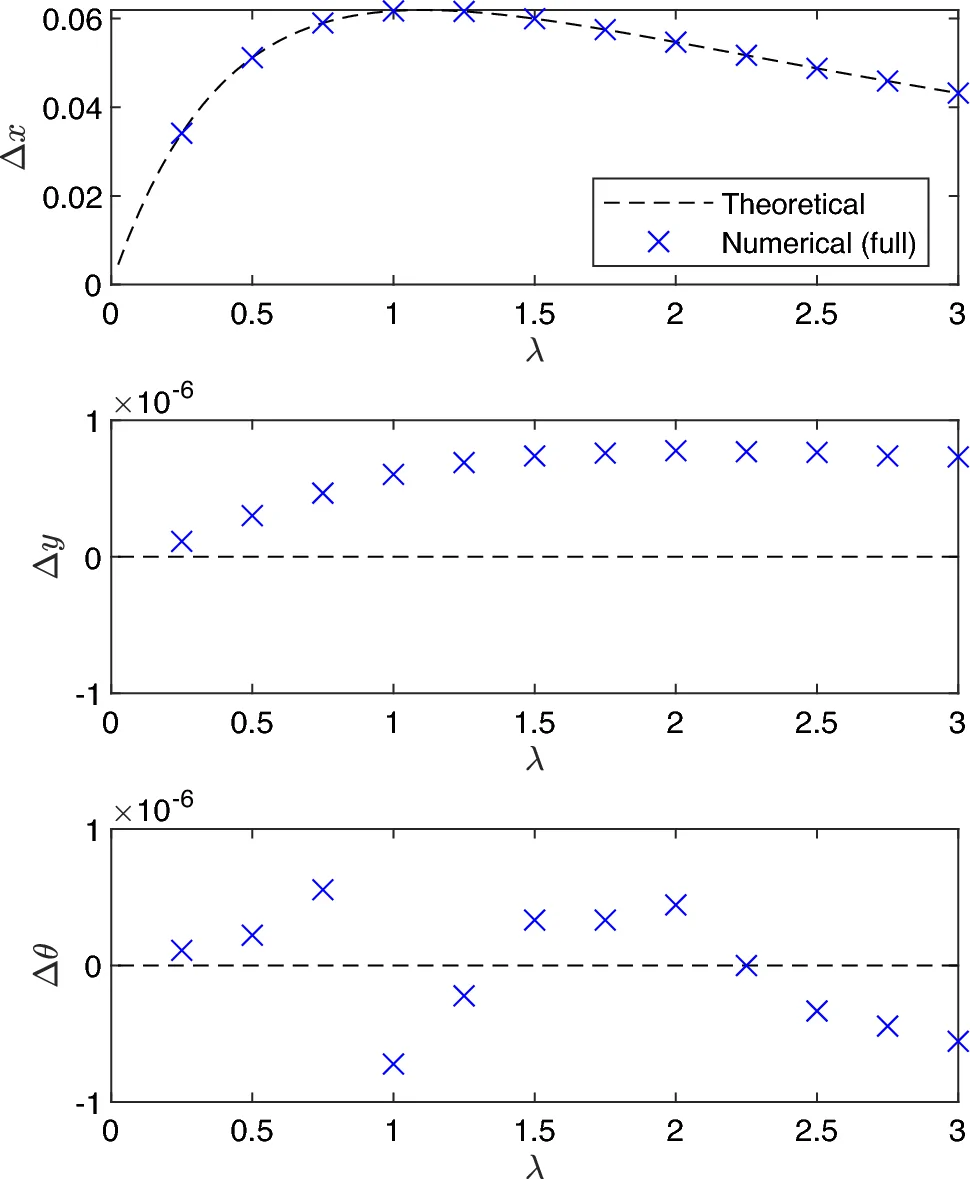
Comparisons between numerically computed and theoretical displacements Δx, Δy, and $\Delta \theta$ as functions of the link length ratio λ for controls corresponding to the vector field $\textbf{h}^3_t$ with amplitude $\xi =10^{-4.5}$. The initial state is $(x_0,y_0,\theta _0,\alpha ^{(-1)}_0,\alpha ^{(1)}_0) = (0,{10^{-2}},0,0,0)$. The common geometrical parameters used are L=1 and $r=10^{-4}$. The full drag coefficients (6) are used for the numerical computations

**Fig. 5:**
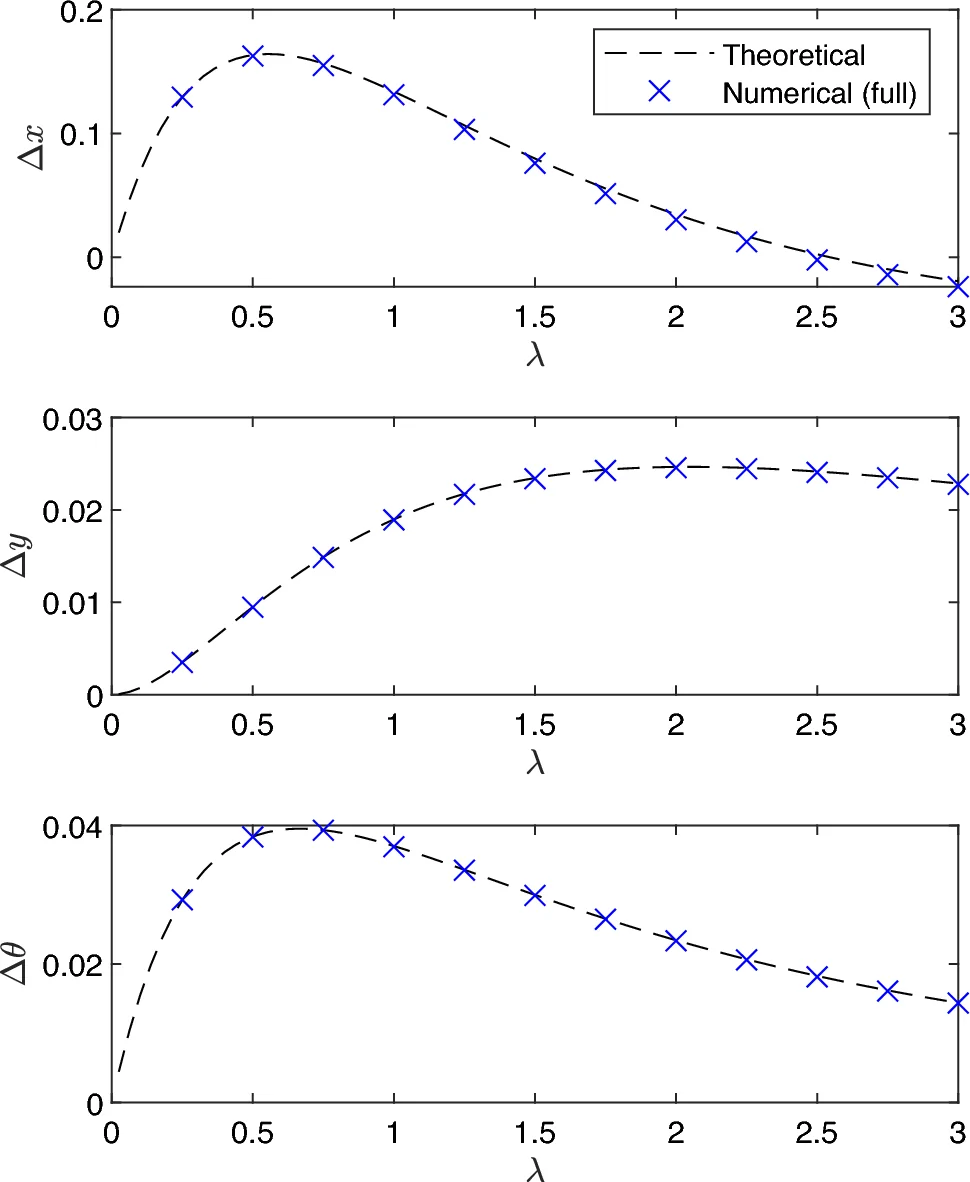
Comparisons between numerically computed and theoretical displacements Δx, Δy, and $\Delta \theta$ as functions of the link length ratio λ for controls corresponding to the vector field $\textbf{h}^4_t$ with amplitude $\xi =10^{-3.5}$. The initial state is $(x_0,y_0,\theta _0,\alpha ^{(-1)}_0,\alpha ^{(1)}_0) = (0,10^{-2},0,0,0)$. The common geometrical parameters used are L=1 and $r=10^{-4}$. The full drag coefficients (6) are used for the numerical computations

To verify the theoretical effects of modifying the ratio λ between the length of the central link and that of the outer links, we numerically solve the control system using the full form of the drag coefficients. As shown in Fig. 4 for $\textbf{h}^3_t$ , the numerical results agree closely with the theoretical prediction based on (14). We note that from the *x*-component of $\textbf{h}_t^3(\lambda )$, it can be determined that the maximum displacement is obtained at $\lambda =(\sqrt{97}-1)/8\approx 1.10611$. We also show the dependence on λ for the vector field $\textbf{h}_t^4$ in (15) and illustrate the numerical results in Fig. 5. The *x*-component of $\textbf{h}_t^4(\lambda )$ is found to be maximized at $\lambda \approx 0.55467$.

**Fig. 6:**
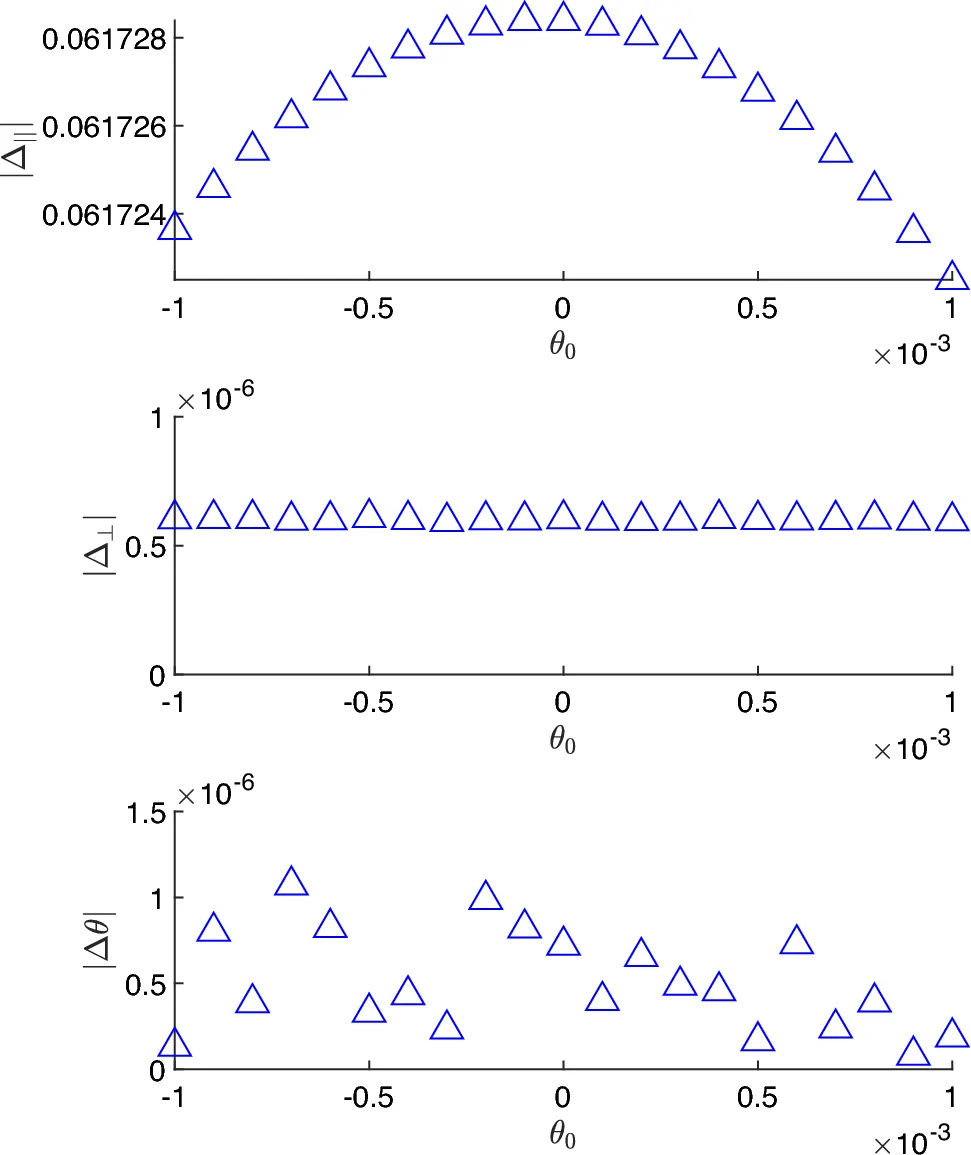
Numerically computed displacements $|\Delta _{\parallel }|$, $|\Delta _{\perp }|$, and $|\Delta \theta |$ in the swimmer parallel, perpendicular, and angular directions as functions of the tilt angle $\theta _0$ with respect to the wall for controls corresponding to the vector field $\textbf{h}^3_t$ . The initial state is $(x_0,y_0,\theta _0,\alpha ^{(-1)}_0,\alpha ^{(1)}_0) = (0,{10^{-2}},\theta _0,0,0)$. The geometrical parameters used are L=1, λ=1, $r=10^{-4}$, and the amplitude parameter is $\xi =10^{-4.5}$. The full drag coefficients (6) are used in these computations

Whereas we only computed the vector fields analytically for the parallel configuration, $\theta _0 = 0$, we show the numerical results for displacements using the piecewise constant controls for $\textbf{h}_t^3$ starting from slightly tilted swimmer configurations in Fig. 6, recalling that the range of admissible tilt angles is restricted by condition (2). In free space, it is known that the $\textbf{h}_t^3$ vector field is used to propel the swimmer along its axis while $\textbf{h}_t^4$ and $\textbf{h}_t^5$ are used to generate perpendicular and rotational motion. Hence, we decompose the displacement into a component parallel to the tilted axis, a component perpendicular to the axis, and an angular component. The parallel component $|\Delta _\parallel |$ appears to have a local maximum at $\theta _0 \approx 0$, however, we note that the variations in $|\Delta _\parallel |$ with $\theta _0$ are comparable to the finite-ξ effects shown in Fig. 2. The perpendicular and angular displacements are negligible, showing that even with the wall, the $\textbf{h}_t^3$ vector field only produces net motion along the axis of the swimmer.

**Fig. 7:**
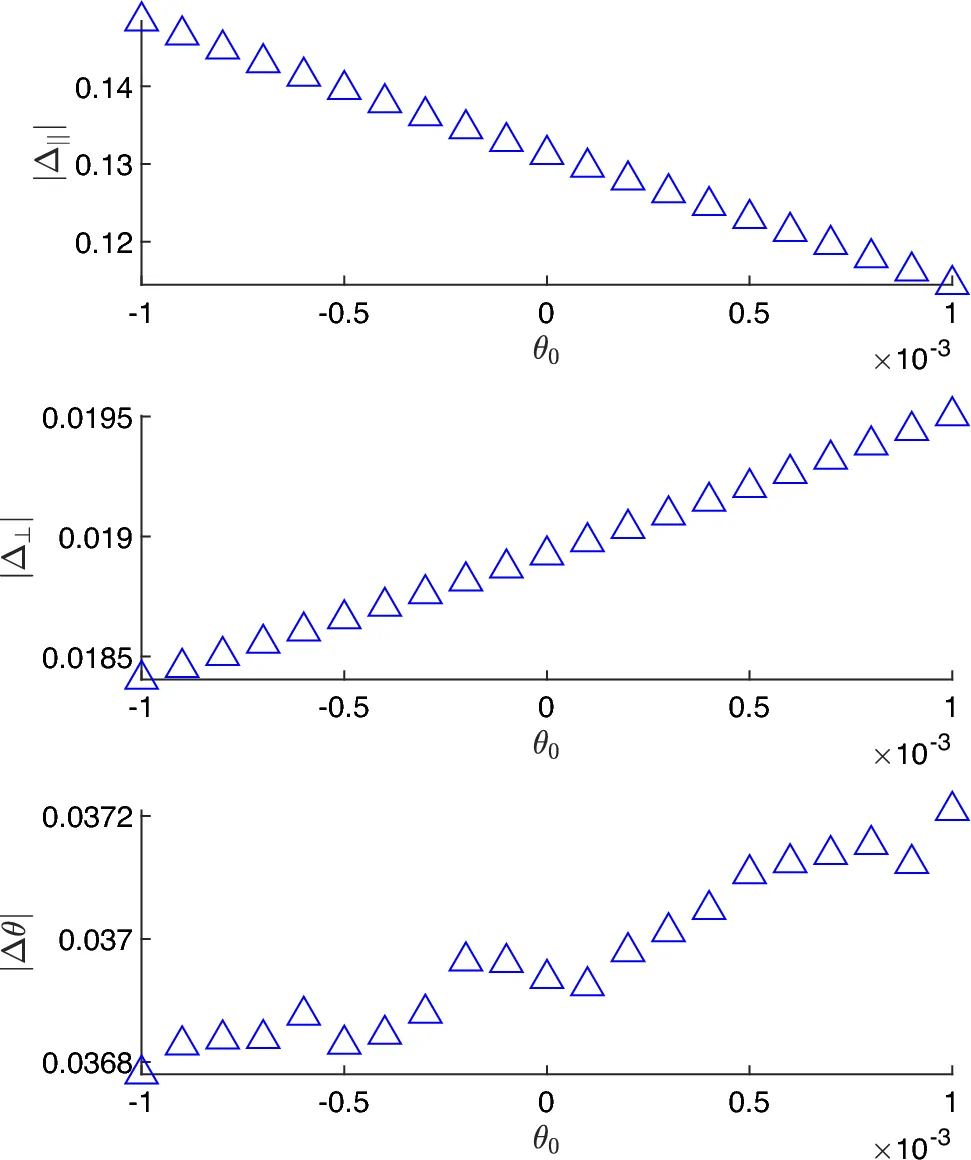
Numerically computed displacements $|\Delta _{\parallel }|$, $|\Delta _{\perp }|$, and $|\Delta \theta |$ in the swimmer parallel, perpendicular, and angular directions as functions of the tilt angle $\theta _0$ with respect to the wall for controls corresponding to the vector field $\textbf{h}^4_t$ . The initial state is $(x_0,y_0,\theta _0,\alpha ^{(-1)}_0,\alpha ^{(1)}_0) = (0,{10^{-2}},\theta _0,0,0)$. The geometrical parameters used are L=1, λ=1, $r=10^{-4}$, and the amplitude parameter is $\xi =10^{-3.5}$. The full drag coefficients (6) are used in these computations

Corresponding analysis of the second-order control in tilted initial configurations is shown in Fig. 7. We find that the parallel displacement decreases approximately linearly and the perpendicular and angular displacements increase approximately linearly with the tilting angle.

## Conclusions

In this work we have investigated the dynamics and controllability of a Purcell three-link swimmer moving through a viscous fluid in a plane perpendicular to a rigid wall. Starting from a wall-corrected resistive-force theory model, we derived an explicit control system valid for configurations that are nearly parallel to the boundary. Using tools from Geometric Control Theory, previous studies proved that the Purcell swimmer is locally controllable in a free-space domain. We now show that the swimmer remains locally controllable in the presence of a no-slip plane wall when it is nearly parallel to the wall, thereby determining that the wall does not hinder controllability. In fact, the wall’s presence has a symmetry-breaking effect and provides a richer structure of the Lie algebra generated by the control vector fields.

All our theoretical predictions are in good agreement with numerical simulations. In particular, we observe that, even though our computation of the brackets $\textbf{h}_t^{i}$ (i=1,…,5) is done using the approximates drag coefficients of the resistive-force theory (see (9)), the theoretical results so obtained are in good agreement with the numerical simulations performed using the completely nonlinear drag coefficients, as Figs. 2–6 show.

Several directions for future research naturally emerge from our analysis. Our treatment of wall effects based on resistive-force theory is a simplification and limited to asymptotic regimes in which $r\ll y_t \ll L_i$ ($i\in \{\pm 1,0\}$). Resistance coefficients valid in other regimes have been proposed (such as $y_t \gg L_i$ and for arbitrary ratios of $y_t/L_i$ ; see [18, 20]). Controllability results could be extended to such regimes by considering more general ratios of the normal and tangential drag coefficients. Retaining higher-order terms in expansion (9a) could also allow one to quantify more precisely the range of validity of the present model.

To overcome the limitations of resistive-force theory, particularly near a wall or in other domain geometries, the swimming trajectories can be computed more accurately using numerical methods such as boundary element methods [12, 28] and regularized Stokeslet segments [19, 29]. Such frameworks would enable higher fidelity simulations for practical applications, though their utility in theoretical proofs may be limited.

Allowing more general three-dimensional motion of the swimmer, several qualitatively new phenomena are expected. First, the configuration space becomes higher-dimensional, with additional orientation degrees of freedom, and the presence of a planar wall breaks rotational symmetry in a non-trivial way. A 3D Purcell-type swimmer near a no-slip boundary could exhibit out-of-plane reorientation, circular trajectories, or stable limit cycles analogous to those observed for helical bacteria near surfaces. From a control perspective, it would be natural to investigate whether controllability persists near configurations parallel to the wall and how the Lie algebra structure is modified by the additional rotational degrees of freedom.

## Data Availability

No datasets were generated or analysed during the current study.

## Footnotes

1 The explicit values of $\sigma _\pm ^{(i)}$ are $\sigma _-^{(-1)}=\sigma _-^{(0)}=\sigma _+^{(-1)}=-1$ and $\sigma _-^{(1)}=\sigma _+^{(0)}=\sigma _+^{(1)}=1$.

2 The explicit values of $\sigma _*^{(i)}$ are $\sigma _*^{(\pm 1)}=1$ and $\sigma _*^{(0)}=-1$. Notice that $\sigma _*^{(i)}=\sigma _-^{(i)}\sigma _+^{(i)}$.
